# Amino Acid Complexed Minerals Zn, Mn, and Cu Improve Bone and Intestinal Characteristics in Laying Pullets

**DOI:** 10.1007/s12011-025-04873-x

**Published:** 2025-11-19

**Authors:** Marcos José Batista dos Santos, Carlos Bôa-Viagem Rabello, Andresa de Gusmão Faria, Waleska R. L. Medeiros-Ventura, Rogério Ventura Silva Junior, Heraldo Bezerra de Oliveira, Fabiano Sellos Costa, Mércia Rodrigues Barros, Alba K. Fireman

**Affiliations:** 1 Animal Science Department, Universidade Federal Rural de Pernambuco, Rua Dom Manoel de Medeiros, s/n, Dois irmãos, Recife, PE 52171-900 Brazil; 2 Veterinary Science Department, Universidade Federal Rural de Pernambuco, Rua Dom Manoel de Medeiros, s/n, Dois irmãos, Recife, PE 52171-900 Brazil; 3 Zinpro Corporation, Eden Prairie, Mn EUA 55344 3 U.S.A.

**Keywords:** Poultry, Trace Elements, Bioavailability Enhancement, Gut Morphology, Hematological Effects

## Abstract

The present study investigates the effect of partially supplemented trace minerals zinc (Zn), manganese (Mn), and copper (Cu) using 2 different sources: inorganic mineral (IM) or amino acid-complexed minerals (AACM), in pullet diets during 6 to 14 weeks. A total of 1120 Lohmann Brown Lite pullets were distributed into 40 experimental cages with 2 dietary treatments. Two dietary treatments were established: a IM supplemented with 70 mg kg⁻^1^ Zn (as oxide), 70 mg kg⁻^1^ Mn (as oxide), and 8 mg kg⁻^1^ Cu (as sulfate), and a test diet (IM + AACM) providing the same total elemental concentrations of Zn, Mn, and Cu, but with 40, 40, and 2.75 mg kg⁻^1^ from IM sources (oxides/sulfate) and 30, 30, and 5.25 mg kg⁻^1^ from AACM, respectively. Data were compared by Student's t-test (*P* < 0.05). Zootechnical performance was not affected by mineral source (*P* > 0.05). However, AACM increased relative intestinal weight (*P* = 0.01) and small intestine length (*P* = 0.05) and decreased cecum length (*P* < 0.01). Tibia calcium and phosphorus contents were 8.5% and 6.9%, respectively, higher (*P* < 0.05) in the AACM group. Birds fed AACM diets showed enhanced tibia bone density (*P* < 0.01). Serum analysis showed elevated (*P* < 0.05) red blood cells, hemoglobin, hematocrit, white blood cells, alkaline phosphatase, albumin, creatinine, and thyroxine levels in birds supplemented with AACM. Supplementation with AACM trace minerals enhance bone, intestine, and blood measurements in laying pullets without impacting growth or organ weights.

**Supplementary Information:**

The online version contains supplementary material available at 10.1007/s12011-025-04873-x.

## Introduction

Laying hens' eggs are a valuable source of protein for human nutrition and serve as raw materials for various industrial products. However, egg farms operate on a narrow profit margin. Therefore, it is essential for pullets to undergo optimal development during the rearing period, maximizing their genetic potential for the laying stage.

While studies on the dietary requirements of laying hens during the production phase have predominantly focused on amino acids, energy, and macro minerals, there is little research concerning trace minerals, especially in pullets. During pullet development, trace minerals such as zinc (Zn), copper (Cu), and manganese (Mn) play essential roles in various aspects of bird development, including body weight and bone development [1–3], intestinal health [4], reproductive organs [5], and immune system [6]. These trace minerals also participate as cofactors in numerous physiological and metabolic processes [7], potentially impacting long-term layer performance.

In commercial pullet diets, the feed ingredients provide trace minerals, however, to meet the birds' total requirements, inorganic minerals (IM) are commonly supplemented, either from oxides or sulfates. This nutritional approach has limitations, as IM sources may dissociate in the gastrointestinal tract and form complexes with antagonistic compounds, reducing their absorption and utilization [8]. These antagonists, such as phytic acid, and fibers are abundant in plant-based feed ingredients [9]. To address these issues, nutritionists have been using organic minerals in animal diets [10].

Organic minerals are formed through the complexation or chelation of dietary minerals with organic ligands. The strength and stability of these molecules depends on factors such as the type of bond formed, the specific metal ion, and the structure of the organic ligand. Common types of organic mineral include those amino acid complexed mineral (AACM), polysaccharide complexes, glycinates and proteinates [11]. The absorption of AACM is mediated by amino acid transporters, reducing competition for mineral absorption sites in the intestine, resulting in higher bioavailability compared to IM forms [8]. Medeiros-Ventura et al. [2] demonstrated that layer-type chickens fed AACM exhibited higher body weight gain, thymus, and cecum weights, superior micromineral deposition in the tibias, and reduced phosphorus (P) excretion compared to IM sources. Furthermore, Burin et al. [6] found that the intestinal morphology and immune function in broilers were superior when birds were fed AACM compared to IM sources. However, research indicates that IM trace minerals, due to their poor bioavailability, may be insufficient to meet the requirements for optimal structural and physiological development in modern poultry [12]. A study by Niknia and Vakili [13] found that Zn-Met not only boosts tibial bone mechanical properties but also preserves eggshell quality, as it implies that Zn-Met could support both skeletal health and eggshell formation.

Moreover, in laying industry, it is common practice to supplement birds with some organic mineral source only during the laying phase, keeping the pullet phase supplemented with IM. This approach aims to minimize costs while providing the birds with both an inexpensive and more bioavailable source, thus ensuring optimal trace mineral nutrition. However, because of the pullets' low feed consumption, investment in highly bioavailable trace minerals is limited. This highlights the importance of investigating the potential benefits of using these minerals. Therefore, this study was carried out to examine supplementation of Zn, Mn, and Cu in pullet diets from IM and AACM sources during the rearing phase on performance, organ weight, bone characteristics, bone and liver mineral deposition, hematology and leucogram, and serum hormones.

## Materials and Methods

### Ethics Statement

The protocol for the development of this study was authorized by the Ethics Committee on Animal Use at the Federal Rural University of Pernambuco (approval no. 064/2016), Also, the experiment was carried out in compliance with the European Union directive no. 2010/63/EU and with the appropriate ARRIVE [14] guidelines for reporting on experiments involving animals.

### Experimental Design and Treatments

A total of 1120 Lohmann Brown Lite pullets, with a mean initial weight of 286 ± 1.00 g, were reared from 6 to 14 weeks of age. The birds were housed in cages measuring 100 × 50 × 50 cm in length, width, and height, respectively. Each cage was equipped with trough feeders, cup drinkers, and ceiling fans throughout the shed. The experiment was conducted in a completely randomized design with 2 treatments and 20 replicates with 28 birds per experimental unit (comprising 2 cages per experimental unit). A housing density of 357 cm^2^/bird was used.

The treatments consisted of 2 diets: one including IM supplementation, and the other being partially supplemented with IM and AACM. The inclusion levels were determined on the basis of IM requirements following the Lohmann Brown Lite manual guideline standards (Table 1). The treatment groups were as follows:

**Table 1:** Calculated and analyzed diets

INGREDIENT, %		6—10 weeks	11—14 weeks
Corn		66.6	68.0
Soybean meal		29.6	28.3
Salt		0.34	0.34
Limestone		1.21	1.21
Dicalcium phosphate		1.45	1.35
Vitamin premix^1^		0.10	0.10
Mineral premix^2^		0.10	0.10
Calsporin BSG^3^		0.05	0.05
Biobond^4^		0.20	0.20
Sodium bicarbonate		0.15	0.15
Dl-methionine 99%		0.07	0.06
Mineral premix		0.10	0.10
Phytase^5^		0.01	0.01
Kaolin		0.02	0.03
Total		100	100
Metabolizable energy, MJ/kg		12.18	11.76
Crude protein, %		18.5	18.0
Crude protein ^6^, %		19.0	18.4
Dry matter ^6^, %		88.5	88.9
Ether extract, %		2.91	2.94
Crude fiber, %		2.93	2.87
Mineral matter, %		5.75	5.57
Calcium, %		1.03	1.00
Phosphorus		0.63	0.63
Available phosphorus, %		0.47	0.45
Sodium, %		0.20	0.20
Lysine, %		0.89	0.86
Methionine, %		0.34	0.32
Methionine + cystine, %		0.59	0.57
Threonine, %		0.63	0.61
Tryptophan, %		0.20	0.20
Arginine, %		1.16	1.12
Isoleucine, %		0.78	0.76
Valine, %		0.79	0.77
Choline, mg/kg		1,239	1,211

^1^Provides per kilogram of product: vit. A, ≥ 8,000,000 IU; vit. D3, ≥ 2,500,000 IU; vit. E, ≥ 6,000 IU; vit. K3, ≥ 1,000 mg; vit. B1, ≥ 1,000 mg; vit. B2, ≥ 4,500 mg; vit. B6, ≥ 2,000 mg; vit. B12, ≥ 12,000 mcg; niacin, ≥ 15 g; calcium pantotenate, ≥ 6,000 mg; folic acid, ≥ 400 mg; biotin, ≥ 25 mg. ^2^Provides per kilogram of product: iodine, 1 mg; selenium, 0.2 mg; iron, 20 mg. ^3^Guarantee levels: Bacillus subtillis: 1 × 1010 cfu g^−1^. ^4^Guarantee levels: sodium and calcium alumino-silicate hydrates: 1000 g kg^−1^. ^5^Quantum blue^®^—ABVista Guaranteed levels: Phytase (min), 10,000 FTU kg^−1^. ^6^Analyzed values

1. IM—supplemented with Zn oxide, Mn oxide, and Cu sulfate at concentrations of 70, 70, and 8 mg kg⁻^1^, respectively.
2. Test diet (AACM)—supplemented with Zn, Mn, and Cu from IM sources at concentrations of 40, 40, and 2.75 mg kg⁻^1^, respectively, in addition to Zn, Mn, and Cu complexed with unspecified essential amino acid at concentrations of 30, 30, and 5.25 mg kg⁻^1^, respectively.

For supplementation with AACM, Zinpro^®^ Availa^®^ ZMC (Zinpro Corp., Eden Prairie, MN, USA) was utilized. Water was provided ad libitum while feed supply was adjusted weekly according to growth requirements. The vaccination program was implemented based on the guidelines outlined in the Lohmann Brown Lite manual. The initial beak trimming procedure was conducted at 4 weeks of age, followed by a second procedure at 11 weeks of age, using an automatic beak trimmer (Avi-Beaker; Wings Ltd., Jandira, Brazil).

Temperature (maximum and minimum) as well as relative humidity data were recorded by a data logger positioned at the center of the facility (Hobo U12-012, Onset Computer Corporation, Bourne, MA, USA), along with 3 digital thermo-hygrometers (7663.02.0.00, Incoterm^®^, Porto Alegre, Brazil) strategically placed in different locations within the premises. The average temperature and air humidity were 26.2 °C and 61%, respectively.

Table 2 presents the calculated and analyzed mineral composition for the experimental diets and water. The analyzed amino acid content of the product is provided in Supplement A. The formulation of the experimental diets followed guidelines, to meet the nutritional requirements of laying birds according to the rearing stage evaluated. To maintain consistency in nutritional content, the diets were formulated to be iso-nutritious, with variations restricted solely to the mineral supplement sources.

**Table 2:** Formulated and analyzed composition of the diets

						6 to 10 weeks				11 to 14 weeks		
		Calculated (mg kg^−1^)				Analyzed^*^ (mg kg^−1^)						
Treatments	Source	Zn	Mn	Cu		Zn*	Mn*	Cu*		Zn*	Mn*	Cu*
IM	IM^1^	70.0	70.0	8.00		92.5	82.1	12.6		105	107	15.5
AACM	IM^1^	40.0	40.0	2.75		93.0	88.7	13.5		95.2	105	15.9
	AACM^2^	30.0	30.0	5.25								

^1^The inorganic (IM) sources of zinc, manganese, and copper were ZnO, MnO, and CuSO_4_; ^2^The zinc, manganese and copper sources in the amino acid complex mineral (AACM) were Availa^®^ Zn, Availa^®^ Mn and Availa^®^ Cu—(Zinpro Corp., Eden Prairie, MN, USA). *The iron (FeSO_4_), Iodine (KIO_3_), and selenium (Na_2_SeO_3_) levels in both experimental diets were 50, 1, and 0.25 mg kg^−1^, respectively

### Performance Variables and Tissue Collection

Birds were individually weighed on the 1 st day of the experiment to determine their average weight and allocate them to the experimental units. Performance variables: body weight, body weight gain (g), feed intake (g), feed conversion ratio (g g^−1^), and uniformity (%), were assessed weekly. Upon reaching 14 weeks, 80 birds (2 per experimental unit) were selected based on their average live weight and euthanized by cervical dislocation, following the guidelines of Resolution N^o^. 1000 from the Brazilian Federal Council of Veterinary Medicine.

Subsequently, the tibiae (both right and left), spleen, liver, pancreas, and intestines were collected. The gizzard and intestines were opened, and their contents were removed. Organs and bones were weighed separately using a semi-analytical scale with a precision of 0.01 g (L3102iH, Bel Engineering^®^, Milan, Italy). Additionally, the whole intestine, small intestine, and cecum were measured using a precision micrometer (iGaging, San Clemente, CA, USA).

### Blood Variables

To assess the hematological profile, 1 bird per replicate was randomly selected in the last week of the experiment for blood sample collection. Four milliliters of blood were collected via jugular venipuncture into a heparin-containing tube. Red blood cells, leukocytes, and platelets were quantified using a Newbauer chamber following dilution with Natt–Herrick reagent. The counting was performed using a microscope (Olympus America CX-41, Center Valley, PA 18034–0610, USA). Hematocrit levels were determined using the microcapillary method, and total serum protein was measured using refractometry. Differential leukocyte counts were obtained by examining slides under an optical microscope (Olympus America CX-41, Center Valley, PA 18034–0610, USA) and staining using the fast panoptic method.

### Serum Biochemistry and Total Protein Concentration

Serum samples were thawed at room temperature (27 °C) and subsequently placed in individual cuvettes according to the Labtest automated biochemical analyzer (Labmax 240 Premium, Japan – Tokyo Boeki Medical System Ltd.) for analysis. Labtest^®^ commercial kits were used for each enzyme and substrate. Aspartate aminotransferase, gamma-glutamyl transferase, alkaline phosphatase, albumin, urea, creatinine, cholesterol, and triglycerides and total protein concentrations were determined.

### Hormonal Profile

The serum samples were kept in 1.5 mL microtubes and stored at −80 ºC. Samples were analyzed for corticosterone, thyroxine, and triiodothyronine. For analysis, the samples were thawed at room temperature (27 °C), homogenized in a vortex, and centrifuged at a rotation speed of 3000 g for 10 min. Subsequently, each sample was placed in individual cuvettes for electrochemiluminescence analysis (Access 2, Beckman Coulter). Samples prepared according to a commercial kit (BIOCLIN^®^) and subsequently read in a spectrophotometer (Bioclin, Biolisa Reader).

### Bone Resistance and Seedor Index

After collection, the tibiae were placed in Falcon tubes clearly labeled for identification and then frozen at −20 °C. The right tibiae were used for mineral composition analysis, while the left tibiae were used for bone strength and density assessment. To investigate bone characteristics, the tibiae was thawed, and the adherent tissues were removed to prevent any harm to the bone structure and air-dried.

Tibial length and weight were measured using a digital caliper (Absolute Digital AOS, Mitutoyo, Brazil; precision of 0.01 mm) and a semi-analytical scale (precision of ± 0.01 g), respectively. The Seedor index [15] was then calculated by dividing the ash weight (mg) by its length (mm). This index serves as an indicator of bone density, with higher values indicating greater density. Bone strength analysis was carried out utilizing a universal texture analyzer (TA-XT Plus, Stable Micro Systems, Surrey, UK) equipped with a 50 kg load cell, operating at a speed of 30 mm min^−1^.

### Computerized Densitometry

**Fig. 1:**
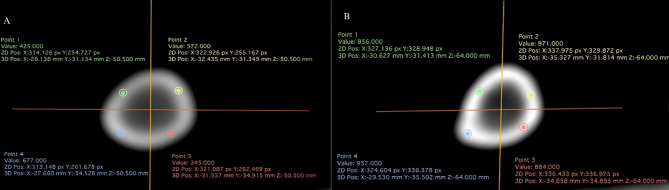
Cross-sectional tomographic image of the middle region of the tibia of chickens showing the regions of interest in the cortical bone to obtain radiodensity values in Hounsfield units (HU). (**A**) Inorganic minerals (IM) treatment. (**B**) Amino acid complexed minerals (AACM) treatment

Bone densitometry was conducted on 40 tibiae (20 replicates per treatment) using a Hi-speed FX1 CT scanner (General Electric, Fairfield, CT, USA). The bones were positioned side by side on the examination table to capture the images. Cross-sectional images were acquired from 2-mm-thick sections with a reconstruction interval of 1 mm, employing 120 kV and an automatic tube current (mA) at a rate of 1 rotation per second. Computed tomography-generated images encompass sectional views that precisely identify the region of interest, facilitating the detection of alterations in bone tissue density (Fig. 1).

The quantitative tomographic imaging yielded bone density values, reflecting the average attenuation of the pixels within the selected region of interest, presented in Hounsfield units. Subsequently, the images were subjected to analysis using Dicom software (version 1.1.7, Horos, Purview, Annapolis, MD, USA) to estimate individual bone radiodensity values at 3 diaphysis section levels (proximal, medial, and distal). Each region was further divided into 4 quadrants, and a circular region of interest was chosen for the densitometric assessment of the cortical bone.

Bone mass densitometry (BMD) results were obtained in Hounsfield units (HU) and subsequently converted to Ca hydroxyapatite (mg/cm^3^) [16, 17].

$$BMD=\frac{200 HUt}{(HUb-HUw)}$$

where Hut is the bone radiodensity measured, Hub is the radiodensity of the bone phantom containing 200 mg of Ca hydroxyapatite/cm^3^, and Huw is the radiodensity of the water phantom without Ca hydroxyapatite.

### Mineral Quantification in Tibia, Liver, and Feed

The bones were oven-dried at 105 °C for 24 h and calcined in a muffle furnace (2000F, Zezimaq, Contagem, Brazil) for 4 h at 600 °C. The livers were weighed, packed individually, properly identified, and frozen at −20 °C. Subsequently, the livers were thawed at room temperature (27 ºC) and oven-dried at 105 °C for 24 h. Feed samples were collected, packed in plastic bags, and frozen at −20 ºC. For analysis, the samples were homogenized, ground in a ball mill, and oven-dried at 105 ºC for 24 h. Approximately 0.5 g of the respective samples were digested in 6 mL nitric acid (65%, AR grade). The tibia samples were digested in an open system, whereas the liver and feed samples were microwave-digested (Mars Xpress, Technology Inside, CEM Corporation, Charlotte, NC, USA) for 30 min. After digestion, the samples were diluted in deionized water, which generated the final volumes of 50, 25, and 25 mL of solution for tibia, liver, and feed, respectively. Mineral quantification was achieved by optical emission spectroscopy (ICP-OES, Optima 7000 DV, Perkin Elmer, Waltham, MA, USA). All amino acids provided per the mineral premixes are shown in Supplement A.

### Statistical Analysis

The normality and homoscedasticity assumptions were tested using the Box-Cox test for analysis of variance. Observations were marked as outliers and excluded from the dataset before statistical analysis when the residual of an observation exceeded the residual mean of the parameter by more than 3 standard deviations (or to reach normality). The variables were subjected to ANOVA, and means were compared using Student's T-test at the 5% significance level. The analysis was conducted using SAS 9.4 software [18].

The statistical model was:

$${\mathrm{Y}}_{\mathrm{ij}} =\upmu + {\mathrm{T}}_{\mathrm{i}} + {\upvarepsilon }_{\mathrm{ij}}$$

where:

- Y_ij_ represents the observed value of the j_th_ experimental unit under the i_th_ treatment.
- μ is the overall mean of the response variable.
- T_i_ is the effect of the i_th_ treatment (i = 1 for IM supplementation, i = 2 for AACM supplementation).
- ε_ij_ is the random error term associated with the j_th_ experimental unit under the i_th_ treatment.

## Results

### Performance

The AACM in the diets provided to the pullets during the growth phase did not influence their body weight (*P* = 0.13), body weight gain (*P* = 0.21), feed intake (*P* = 0.51), feed conversion ratio (*P* = 0.55), or uniformity (*P* = 0.30), as indicated in Table 3.

**Table 3:** Mean values of body weight, Body weight gain (BWG), Feed intake (FI), feed conversion ratio (FCR), and uniformity of 14-week-old laying pullets fed 2 different sources of Zn, Mn and Cu

Treatments	Variables				
	Body weight	BWG	FI	FCR	Uniformity
	(g)	(g)	(g)	(g g^−1^)	(%)
IM^1^	1,047	726	2,675	3.642	71.0
AACM^2^	1,032	734	2,698	3.634	74.0
Means	1,040	732	2,686	3.631	73.0
*P-*value	0.134	0.206	0.514	0.547	0.299
SEM	4.99	4.59	6.91	0.024	1.30

^1^IM = Inorganic minerals at 70, 70, and 8 mg kg^−1^ of Zn, Mn, and Cu, respectively

^2^AACM = Amino acid complexed minerals at 40, 40, and 2.75 mg kg^−1^ of Zn, Mn, and Cu, respectively, plus inorganic minerals at 30, 30, and 5.25 mg kg^−1^ of Zn, Mn, and Cu, respectively

SEM: Standard error of the mean

### Organs

Table 4 shows the yield of the thymus, bursa of Fabricius, spleen, pancreas, liver, and whole intestines of 14-week-old laying pullets fed 2 different sources of Zn, Mn, and Cu. The mineral source did not affect the relative weight of the thymus (*P* = 0.49), bursa of Fabricius (*P* = 0.34), spleen (*P* = 0.25), pancreas (*P* = 0.16), and liver (*P* = 0.20). Supplementation with AACM in the diet resulted in a higher relative intestinal weight (*P* = 0.01).

**Table 4:** Yield of thymus, bursa of Fabricius, spleen, pancreas, liver, and whole intestines of 14-week-old laying pullets fed 2 different sources of Zn, Mn and Cu

Treatments	Thymus	Bursa*	Spleen*	Pancreas	Liver	Intestine
	(%)					
IM^1^	0.430	0.170	0.260	0.250	2.44	5.29^b^
AACM^2^	0.410	0.180	0.280	0.270	2.34	5.57^a^
Means	0.420	0.170	0.270	0.260	2.39	5.43
*P*-value	0.489	0.341	0.249	0.158	0.196	0.014
SEM	0.018	0.009	0.006	0.005	0.04	0.06

^1^IM = Inorganic minerals at 70, 70, and 8 mg kg^−1^ of Zn, Mn, and Cu, respectively

^2^AACM = Amino acid complexed minerals at 40, 40, and 2.75 mg kg^−1^ of Zn, Mn, and Cu, respectively, plus inorganic minerals at 30, 30, and 5.25 mg kg^−1^ of Zn, Mn, and Cu, respectively

SEM: Standard error of the mean

^a, b^ Means without a common superscript letter differ based on Student’s t-test (*P* < 0.05)

* Box-Cox transformation: bursa^0.5^; spleen^0.75^

**Fig. 2:**
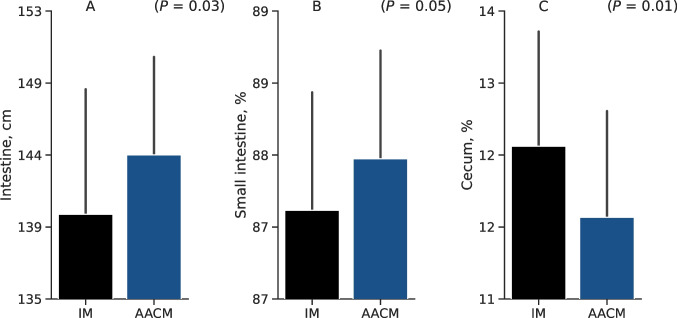
Whole intestine length (**A**), relative small intestine (**B**), and relative cecum (**C**) of 14-week-old laying pullets fed 2 different sources of Zn, Mn, and Cu. IM = Inorganic minerals at 70, 70, and 8 mg kg^−1^ of Zn, Mn, and Cu, respectively. AACM = Amino acid complexed minerals at 40, 40, and 2.75 mg kg^−1^ of Zn, Mn, and Cu, respectively, plus inorganic minerals at 30, 30, and 5.25 mg kg-1 of Zn, Mn, and Cu, respectively. Data are presented as means ± SD. P-value ≤ 0.05 differs from Student’s t-test. The relative lengths are correlated to the whole intestine.

Specifically, intestine length (*P* = 0.03), relatively small intestine lengths were greater (*P* = 0.05) in AACM-fed pullets compared to IM-fed groups. In contrast, the relative cecum length was higher (*P* < 0.01) in the IM-supplemented hens (Fig. 2).

### Minerals in Tibia

Quantification of minerals in the tibia demonstrated that Zn (*P* = 0.37), Mn (*P* = 0.08), and Cu (*P* = 0.95) were not influenced by the trace mineral supplementation (Table 5). However, Ca and P contents were higher (*P* < 0.01) by 8.5% and 6.9%, respectively, in the bones of birds supplemented with AACM. Similarly, the deposition of Zn (*P* = 0.80), Mn (*P* = 0.17), Cu (*P* = 0.80), Ca (*P* = 0.31), and P (*P* = 0.13) in the liver was not affected by the source of mineral supplementation (Table 5).

**Table 5:** Bone and liver mineral composition of 14-week-old laying pullets fed 2 different sources of Zn, Mn, and Cu

Treatments	Bone mineral composition				
	Zinc	Manganese	Copper	Calcium	Phosphorus
	(mg kg^−1^)			(g kg^−1^)	
IM^1^	262	5.55	5.60	445^b^	231^b^
AACM^2^	271	6.01	5.62	486^a^	247^a^
Means	266	5.80	5.61	466	239
*P*-value	0.375	0.076	0.951	< 0.001	< 0.001
SEM	4.95	0.13	0.16	5.59	2.53
	Liver mineral composition				
IM^1^	102	10.5	19.3	0.510	12.6
AACM^2^	101	9.80	19.1	0.530	12.2
Means	101	10.2	19.2	0.520	12.4
*P*-value	0.803	0.165	0.797	0.312	0.128
SEM	0.12	0.001	0.001	0.230	0.15

^1^IM = Inorganic minerals at 70, 70, and 8 mg kg^−1^ of Zn, Mn, and Cu, respectively

^2^AACM = Amino acid complexed minerals at 40, 40, and 2.75 mg kg^−1^ of Zn, Mn, and Cu, respectively, plus inorganic minerals at 30, 30, and 5.25 mg kg^−1^ of Zn, Mn, and Cu, respectively

SEM: Standard error of the mean.

^a, b^Means without a common superscript letter differ based on Student’s t-test (*P* < 0.05).

### Bone Characteristic

The bone characteristic results (Table 6) showed no differences based on the source of supplemental Zn, Mn, and Cu in the experimental diets on dry matter (*P* = 0.38), tibial weight (*P* = 0.36), tibial length (*P* = 0.82), bone resistance (*P* = 0.74), or Seedor index (*P* = 0.34). Proximal, medial, distal, and mean bone density were higher (*P* < 0.01) in the pullets provided with AACM diets.

**Table 6:** Bone variables and densitometry of 14-week-old laying pullets fed 2 different sources of Zn, Mn, and Cu

	Bone variables					Bone densitometry			
Treatments	DM	Weight	Length	BR	SI	Proximal	Medial	Distal	Mean
	(%)	(g)	(cm)	(kgf∕cm^2^)	(mg mm^−1^)	(mg∕cm^3^ Ca hydroxyapatite)			
IM^1^	62.9	9.06	109	19.6	82.7	332^b^	524^b^	376^b^	411^b^
AACM^2^	61.9	9.22	110	19.3	84.1	518^a^	1349^a^	1037^a^	954^a^
Means	62.4	9.14	109	19.4	83.4	425	937	698	660
*P*-value	0.377	0.363	0.818	0.739	0.379	< 0.001	< 0.001	< 0.001	< 0.001
SEM	0.51	0.09	0.53	0.52	0.75	18.5	68.1	56.1	45.7

^1^IM = Inorganic minerals at 70, 70, and 8 mg kg^−1^ of Zn, Mn, and Cu, respectively

^2^AACM = Amino acid complexed minerals at 40, 40, and 2.75 mg kg^−1^ of Zn, Mn, and Cu, respectively, plus inorganic minerals at 30, 30, and 5.25 mg kg^−1^ of Zn, Mn, and Cu, respectively

SEM: Standard error of the mean

^a, b^ Means without a common superscript letter differ based on Student’s t-test (*P* < 0.05)

DM: dry matter; BR: bone resistance; SI: Seedor index

### Blood Variables

Analysis of blood variables (Table 7) showed that pullets fed AACM had higher levels of red blood cells (*P* = 0.03), and hemoglobin (*P* = 0.02), compared to the control group. Trace mineral supplementation did not affect mean corpuscular volume (*P* = 0.15), mean corpuscular hemoglobin (*P* = 0.36), hematocrit (*P* = 0.06), complete blood count (*P* = 0.64), total white blood cell count (*P* = 0.06), total protein (*P* = 0.52), fibrinogen (*P* = 0.47), heterophils (*P* = 0.12), eosinophil (*P* = 0.06), basophils (*P* = 0.62), lymphocytes (*P* = 0.64), or monocytes (*P* = 0.16).

**Table 7:** Blood variables and leukogram of 14-weeks-old pullets fed 2 different sources of Zn, Mn, and Cu

Treatments	Blood Count						
	RBC	HGB	HTC	MCV	MCH	CBC	WBC
	(10^6^/mm^3^)	(%)	(%)	(fL)	(%)	(/mm^3^)	(10^3^/mm^3^)
IM^1^	2.67^b^	10.7^b^	33.0	122	32.0	34.1	14.3
AACM^2^	3.05^a^	11.2^a^	34.8	116	32.3	28.5	16.6
Means	2.84	10.9	33.9	119	32.2	31.6	15.3
*P*-value	0.027	0.022	0.059	0.146	0.363	0.140	0.056
SEM	0.08	0.13	0.41	2.39	0.14	1.40	0.64
**Treatments**	**T. Proteins**	**Fibrinogen**	**Leukogram**				
			**Heterophils**	**Eosinophils**	**Basophils**	**Lymphocytes**	**Monocytes**
	(g dL^−1^)	(mg dL^−1^)	(10^3^/mm^3^)				
IM^1^	4.84	213	9.33	0.840	0.060	8.70	1.50
AACM^2^	5.03	185	12.7	1.52	0.100	10.8	2.39
Means	4.68	200	11.0	1.18	0.080	9.9	1.94
*P*-value	0.517	0.469	0.116	0.065	0.615	0.643	0.164
SEM	0.07	28.1	0.37	0.075	0.038	0.397	0.016

^1^IM = Inorganic minerals at 70, 70, and 8 mg kg^−1^ of Zn, Mn, and Cu, respectively

^2^AACM = Amino acid complexed minerals at 40, 40, and 2.75 mg kg^−1^ of Zn, Mn, and Cu, respectively, plus inorganic minerals at 30, 30, and 5.25 mg kg^−1^ of Zn, Mn, and Cu, respectively

SEM: Standard error of the mean

^a,b^Means without a common superscript letter differ based on Student’s t-test (*P* < 0.05)

RBC = Red blood cell; HGB = Hemoglobin; HCT = Hematocrit; MCV = Mean Corpuscular Volume; MCH = Mean corpuscular hemoglobin; CBC = Complete blood count; WBC = White blood cell; T. Proteins = Total proteins

**Fig. 3:**
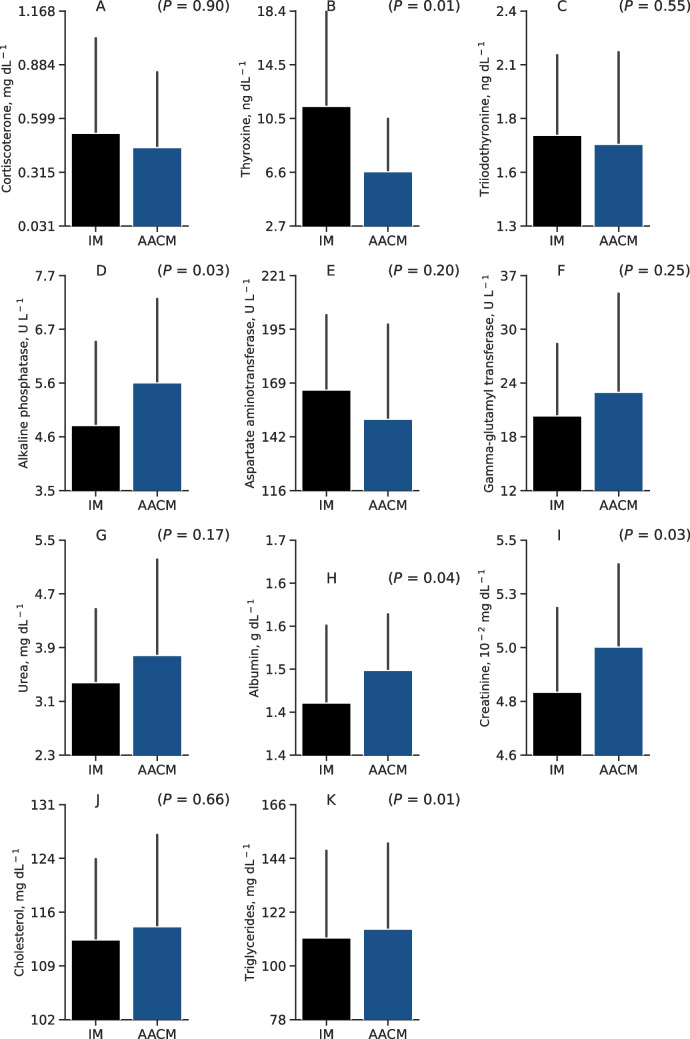
Corticosterone (**A**), thyroxine (**B**), triiodothyronine (**C**), alkaline phosphatase (**D**), aspartate aminotransferase (**E**), gamma-glutamyl transferase (**F**), urea (**G**), albumin (**H**), creatinine (**I**), cholesterol (**J**), and triglycerides (**K**) of 14-week-old laying pullets fed 2 different sources of Zn, Mn and Cu. IM = Inorganic minerals at 70, 70, and 8 mg kg^−1^ of Zn, Mn, and Cu, respectively. AACM = Amino acid complexed minerals at 40, 40, and 2.75 mg kg^−1^ of Zn, Mn, and Cu, respectively, plus inorganic minerals at 30, 30, and 5.25 mg kg^−1^ of Zn, Mn, and Cu, respectively. Data are presented as means ± SD. *P*-value ≤ 0.05 differs from Student’s T-test of phytase enzymes

Analysis of the serum biochemical profile (Fig. 3) showed that birds supplemented with IM had higher thyroxine hormone (*P* = 0.01) compared to the AACM group. In contrast, serum concentrations of alkaline phosphatase (*P* = 0.03), albumin (*P* = 0.04), creatinine (*P* = 0.03), and triglycerides (*P* = 0.01) were higher in birds fed supplementation with AACM. However, the sources did not influence corticosterone (*P* = 0.90), triiodothyronine (*P* = 0.55), aspartate aminotransferase (*P* = 0.20), gamma-glutamyl transferase (*P* = 0.25), urea (*P* = 0.17), and cholesterol (*P* = 0.66).

## Discussion

Supplementing pullet diets with AACM increased intestinal weight and length, tibial Ca and P content, bone density, red blood cell counts, hemoglobin, alkaline phosphatase, albumin, and creatinine, compared to IM supplementation. Such improvements imply improved systemic responses in pullets receiving partial replacements of IM with AACM.

Irrespective of the source, the performance of the birds was not affected by the trace minerals. Body weight beyond the recommended could lead the birds to accelerate the laying cycle or even accumulate lipids in the body [19]. The accelerated onset of lay in heavy birds puts pressure on the oviduct before it is fully developed and prepared for egg production. Meanwhile, excessive fat deposition in the abdomen can physically push and displace the oviduct [20]. The birds in this study were within the body weight recommendations from the Lohmann Brown Lite management guideline manual. Meeting these growth standards indicates the trace mineral supplements did not cause excessive gains.

Medeiros-Ventura et al. [2] found no differences in performance when supplementing layer-type chickens with AACM or IM trace minerals from 1 to 30 days of age, consistent with our observations that trace mineral source did not impact these performance variables in pullets. However, even with no differences in performance, the trace mineral supplements may still have influenced aspects of reproductive physiology [5]. Stated that when supplementing birds with AACM from 16 to 25 weeks, there is an acceleration in the egg layer. Nonetheless, birds receiving AACM had a heavier oviduct and reached 50% of the egg production 2 days before the IM group.

The addition of phytase enzymes and adsorbents to feed formulations can significantly affect mineral dynamics in poultry nutrition. Adsorbents have been found to bind to free minerals in the gastrointestinal tract, potentially making them insoluble and less accessible in the intestinal lumen [21]. Additionally, adding phytase to diets high in these insoluble minerals (IM) may worsen the development of insoluble complexes and increase competition for absorption sites in the intestinal epithelium. This is mostly due to phytase's hydrolysis of phytate, which releases not only phytic phosphorus but also various trace minerals [22]. However, including AACM in poultry diets may help to reduce some of these issues. The stability of AACM increases mineral bioavailability and reduces interactions with diet components that may otherwise limit absorption [23]. The potential binding effects of adsorbents and competitive interactions resulting from phytase activity did not appear to have a substantial influence on AACM supplementation in this study. These findings imply that the inclusion of AACM in poultry diets may be a viable method for maintaining appropriate mineral status, even under conditions that generally reduce mineral bioavailability.

The length of the intestine is closely associated with enhanced nutrient absorption, as a longer intestine offers a greater surface area and increased crypt count for optimal nutrient uptake. Within the intestinal lining, versatile stem cells of crypts transform into absorptive enterocytes, mucus-secreting goblet cells, and enteroendocrine cells [24]. A longer intestine, combined with a higher crypt count, enhances the pool of enterocytes available for higher digestion and absorption of dietary nutrients. This extended absorptive capacity, achieved through a greater intestinal length and crypt count, supports elevated dietary utilization, and bird development [25].

The AACM supplementation promoted intestinal growth in laying hens, however, multiple factors influence gastrointestinal development, including feeding intake, diet composition, and the intestinal microbiome [26]. The dynamic interaction between diet and microbiota can significantly affect intestinal maturation [27–29]. The enhanced development of the intestine from the birds supplemented with AACM diets in this study, is related to the weight and size of the duodenum and jejunum. The anti-inflammatory effects of Zn from AACM sources likely contributed to the enhanced development of the intestinal tract in birds fed these diets. Through several mechanisms, including reducing the activation of NF-κB and its target genes like TNF-α and IL-1, Zn has been demonstrated to have inflammation-modulating effects [30]. Also, Zn has been demonstrated to enhance the cytokine production of Th1 cells, reduce the production of inflammatory cytokines, and decrease oxidative stress [31]. The higher bioavailability of Zn-AACM compared to Zn as ZnO, aids in regulating inflammatory pathways and promoting epithelial cell proliferation. This element acts in connections in epithelial cells within the gastrointestinal tract, contributing to the integrity of the tight junctions when faced with challenges and reducing the likelihood of leaky gut and inflammatory responses [32]. This phenomenon improved the growth and functionality of the duodenum and jejunum as Zn operates through distinct Zn-dependent proteins or transcriptional regulators such as NF-kB and A20 protein modulation [33, 34].

The reduced cecum length observed in birds fed AACM is likely related to microbiome changes caused by lower levels of Zn, Mn, and Cu. With decreased levels of these minerals available to nourish microbial populations, the growth and activity of certain microbes were potentially inhibited [35]. This microbiome modulation resulted in less fermentation and bacterial biomass accumulation in the cecum, which could explain the shorter cecum length observed. Our findings are in accordance with Medeiros-Ventura et al. [2], who found that the inclusion of Zn, Mn, and Cu complexed with amino acids in the starter phase (1–30 days-old) of laying chicks significantly changed the size and weight of the birds' cecum. In addition, Santos et al. [1] reported that supplementing laying hens from 78 to 98 weeks with AACM resulted in better villus height and absorptive area in the duodenum, as well as a higher villus:crypt ratio in the jejunum, compared with birds supplemented with chelated glycinate. Additionally, Santos et al. [10] reported that a 40% reduction in the recommended levels of Zn, Mn, Cu, Fe, Se, and I significantly improved performance, resulting in thicker eggshells and enhanced tibial bone density (*P* < 0.05). These benefits were particularly pronounced at lower supplementation levels, enhancing nutrient absorption and overall intestinal health.

Furthermore, supplementing poultry feed with Cu can yield advantageous effects on the digestive system. This alteration in the intestinal microbiome could enhance the integrity of the intestinal lining, consequently resulting in improved nutrient absorption [36]. Copper is recognized for its antimicrobial properties, capable of influencing the microbial balance within the gut [37]. Moreover, Cu might contribute to the reduction of intestinal inflammation by decreasing the accumulation of immune cells [38]. Grande et al. [4] demonstrated that broilers supplemented with Zn complexed with amino acids exhibited a reduction in harmful bacterial populations, particularly Clostridium spp., compared to birds supplemented with IM. Furthermore, it is plausible that the Bacillus subtilis present in the dietary probiotic in this study may have benefited from the reduced Clostridium population, as these 2 bacterial genera exhibit competitive exclusion [39]. This antagonistic relationship between Bacillus and Clostridium suggests that the suppression of one genus may facilitate the proliferation of the other, potentially enhancing the probiotic effects of B. subtilis in the gastrointestinal tract of broilers.

The higher content of Ca and P in the tibia of the birds supplemented with AACM might be attributed to reduced competition for transport proteins in the intestinal lumen, as these elements share the same transport channels as Mn and Zn. The AACM is absorbed through the amino-acid transport system, leading to improved absorption efficiency for supplemented Zn, Mn, and Cu [8, 10, 40]. This decreased competition with transporters also utilized by Ca and P, such as Divalent-metal transporter-1 (DMT1) and Zinc transporter-4 (ZIP4) [41], enables more efficient absorption of these minerals. With reduced competition from the supplemented minerals, more dietary Ca and P can be transported via these shared pathways into circulation and deposited in the tibia bone. This indicates that AACM enhances mineral bioavailability by modulating competitive inhibitory dynamics during intestinal absorption. In a study by Medeiros-Ventura [2], supplementing layer-type pullets from 1 to 30 days did not yield a statistical difference in Ca and P mineral bone deposition between IM and AACM. This observation could be attributed to the young age of the pullets, as peak bone growth occurs between the fourth and the fifteenth week [42]. Additionally, the relatively short supplementation period may not have allowed enough time for potential differences in tibial mineralization between IM and AACM to become evident.

Neither IM nor AACM supplementation affected Zn, Mn, and Cu in the tibias or liver of the birds. This lack of influence is likely attributed to the sufficient levels of these minerals already present in the diets, considering that additional supplementation with IM or AACM did not lead to increases in the deposition of these minerals within the measured tissues. Although the mechanisms of trace mineral deposition from AACM remain unknown, further investigation is necessary for a better understanding of these processes.

Regarding bone traits, supplementing with trace mineral sources did not result in significant differences in average breaking strength or Seedor index, regardless of the source. However, birds that received the AACM supplement displayed greater tibial radiodensity. This implies that the conventional methods used to measure breaking strength and Seedor index may not adequately reflect true differences in bone quality among mineral sources. More advanced techniques that assess bone composition and microstructure, beyond mechanical properties alone, might be necessary to fully understand the effects of diverse mineral supplements. While breaking strength and Seedor index offer convenient measurements, they fail to capture the nuanced aspects of bone structure and chemistry that could account for the observed increase in tibia mineralization due to AACM supplementation. In contrast, bone tissue density serves as a biophysical measure that offers insights into bone's structural characteristics and estimates the mineral concentration per unit of bone volume [1, 43]. This metric provides valuable context for interpreting results, aiding in clarifying the increased density of bone identified through bone densitometry in this study. The Seedor index offers a whole-bone metric for ash content relative to length, which is a useful general measurement, however, CT densitometry provides a much more granular, regional, three-dimensional assessment of mineral concentration, specifically within defined cortical bone volumes. The fact that the densitometry results showed a significant increase in the medial region (157% increase; *P* < 0.01) while the Seedor indices remained statistically similar (IM: 82.7 vs. AACM: 84.1 mg/mm; *P* = 0.38) it implies that AACM supplementation preferentially increased cortical bone mineralization in the diaphysis without causing a proportional change in the total bone ash. Onyango et al. [43] showed that densitometry methods can detect localized mineral changes that whole-bone measurements, like the Seedor index, are likely to miss. The heightened bone density observed in pullets supplemented with AACM infers an enhanced skeletal structure as the birds approach the laying period. This improvement might contribute to the prevention of bone lesions [44] and promote the laying of eggs with thicker eggshells [12, 45].

In young pullets, long bones grow through endochondral ossification, where proliferating chondrocytes are replaced by osteoblasts forming trabecular bone [46]. With surging estrogen, osteoblasts switch to forming medullary bone, a woven, labile Ca source for eggshells [47]. Bone is a complex tissue with organic (collagen, proteoglycans) and inorganic (Ca, P) components interacting to provide elasticity, strength and fracture resistance based on their ratio. While minerals such as Zn, Mn, and Cu are a small fraction of bone, they are essential for metabolism. The mechanical properties of bone tissue like elasticity, strength, and stiffness arise from the interaction between the organic and inorganic components during matrix formation [48–51].

Enzymes containing Mn and Cu, including glucuronyltransferases and lysyl oxidases, play a role in forming the collagen matrix in bones [52, 53]. Additional studies have shown that dependent enzymes, such as carbonic anhydrase and phosphatases, are important for bone formation and resorption processes [54]. Insulin-like growth factor-I (IGF-I), which promotes growth hormone release and is regulated by dietary Zn intake, has been found to be essential for longitudinal bone growth [55, 56]. Roughead et al. [56] also reported that Cu intake affects IGF-I levels. Furthermore, Mn deficiency may lead to perosis, which involves joint abnormalities and enlarged long bones [57].

Regarding blood variables, leukogram, and serum hormones, the AACM diets exhibited higher red blood cell counts, and hemoglobin levels. The observed increases in red blood cell count (14.2%) and hemoglobin concentration (4.7%) are well within the accepted physiological ranges for laying pullets, pointing toward enhanced erythropoiesis rather than any sign of pathological erythrocytosis. Similar hematological shifts have been previously identified in pullets as part of normal breed and developmental variations. Such modest improvements in the blood’s oxygen-carrying capacity have noteworthy implications. For example, even small increments in hemoglobin can improve tissue oxygenation and metabolic output, a pattern observed in studies of both embryonic and postnatal avian erythropoiesis [58, 59]. This increase may have occurred due to the elevated circulating levels of trace minerals (Zn and Cu) within the birds' bodies. Zinc plays a crucial role in synthetizing new red blood cells from the bone marrow, and serum Zn levels have been positively correlated with hemoglobin, red blood cell counts, and hematocrit levels [60]. Additionally, studies have indicated a significant positive correlation between Cu and red blood cell counts in relation to different hematological variables and blood levels [61]. Copper is required for ceruloplasmin activity, which facilitates the conversion of ferrous Fe into its ferric form for binding to transferrin and subsequent transport to the bone marrow [62], highlighting its significance. The elevated means of hematological variables observed in birds fed AACM diets indicate that these trace minerals were provided in bioavailable forms and were increased enough to optimize erythropoiesis and hemoglobin synthesis.

Birds fed AACM or IM did not exhibit a statistical difference in immune organs. However, AACM showed higher levels of white blood cells in contrast to IM forms. Although assessments of the weights of the immune organs were similar, the amino acid complexed trace minerals may have improved certain elements of immune function, such as white blood cell generation and activity. Trace minerals are crucial for the development, maturation, and effector activities of lymphocytes, neutrophils, and other white blood cells [63], and in comparison to IM forms, AACM may increased the bioavailability of Zn, Cu, and Mn. White blood cells contain elevated concentrations of Zn, which is essential for the development and activation of these immune cells [5, 64]. In this study, the white blood cell counts were not statistically different. A deficiency in Zn can lead to decreased lymphocyte numbers and reduced immune function [64]. Similar to Zn, neutrophils have high levels of Cu, which participates as a co-enzyme, helping generate free radicals that neutrophils use to eliminate engulfed bacteria [65]. The interrelationship between trace minerals and white blood cells highlights the importance of ensuring an adequate dietary intake of micronutrients such as Zn and Cu, and supplementation with AACM may provide bioavailable forms of these minerals to support white blood cell development and activity. Pereira et al. [5], who supplemented layer hens with AACM and IM from 17 to 25 weeks, found higher values for white blood cells, heterophils, and eosinophils in birds fed AACM.

Compared to the control group, the serum of birds given Zn, Mn, and Cu as AACM displayed elevated levels of alkaline phosphatase, albumin, creatinine, and triglycerides, while birds fed mineral salts in IM form exhibited higher concentrations of thyroxine. The higher mineralization in the bones of the birds could be associated with elevated levels of alkaline phosphatase. This association is due to the enzyme's role in bone development by modulating mineralization. Specifically, it acts to hydrolyze inhibitors of mineral formation and generates inorganic phosphate, both of which promote the growth of hydroxyapatite crystals in bone tissue [66]. Beyond its role in the skeletal system, intestinal alkaline phosphatase helps regulate dietary Ca absorption. This is achieved by dephosphorylating phospholipids from feed, making Ca more bioavailable for absorption [67]. Zinc appears to have a significant impact on alkaline phosphatase [68].

Birds receiving the AACM diet showed lower thyroxine concentrations than those fed IM, a difference that seems consistent with reduced metabolic activity and slower protein turnover, however, the decline in thyroid hormones may also help explain why intact albumin levels were higher in circulation, given the role of these hormones in protein breakdown [69]. Still, the connection is not entirely known, and the central mechanisms remain uncertain. In addition, serum protein levels appear to interconnect with mineral balance, particularly calcium and phosphorus. Since a measurable fraction of circulating calcium is bound to proteins, shifts in protein concentrations can directly influence how much free calcium is present in the blood [70].

In the current study, tibia bone Ca and P levels and albumin levels were significantly higher in AACM-fed birds than in IM-fed birds. This implies a possible relationship between elevated circulating albumin and accelerated bone mineralization. The minerals Ca and P have been embedded more extensively in the bones of AACM-fed birds, implying that there may be more of these minerals in the blood as well. Through the binding of extra circulating Ca, it is possible to connect the enhanced albumin levels in these birds to their increased bone mineral content.

## Conclusions

The supplementation of AACM in the diets of Lohmann Brown Lite pullets from 6 to 14 weeks of age did not significantly affect growth performance or organ weights. However, it led to higher intestinal length and weight, increased bone mineral content and density, and influenced blood variables and serum biochemistry profiles. These findings highlight the potential of AACM as a trace mineral source to improve bone health and physiological responses in laying hen type pullets.

## Supplementary Information

Below is the link to the electronic supplementary material.

Supplementary file1 (DOCX 18 KB)

## Data Availability

The data used in this study are available from the corresponding author on reasonable request.
