# Supplementary material for: Amino Acid Complexed Minerals Zn, Mn, and Cu Improve Bone and Intestinal Characteristics in Laying Pullets
Source: Biol Trace Elem Res. 2025 Nov 19;204(5):3037–51. doi: 10.1007/s12011-025-04873-x (PMC13149724; doi:10.1007/s12011-025-04873-x)
Supplement: Supplementary file 1 — Supplementary file1 (DOCX 18 KB) [file 12011_2025_4873_MOESM1_ESM.docx]

Supplement A. Amino acid content (% of the feed) of Cu, Mn, and Zn complexed to amino acids

| **Amino Acid** | **Availa - Zn** | **Availa - Mn** | **Availa - Cu** |
| --- | --- | --- | --- |
| Alanine, % | 0.100 | 0.183 | 0.004 |
| Arginine, % | 0.088 | 0.169 | 0.004 |
| Aspartic Acid, % | 0.106 | 0.197 | 0.004 |
| Glutamic Acid, % | 0.213 | 0.408 | 0.008 |
| Glycine, % | 0.119 | 0.225 | 0.005 |
| Histidine, % | 0.013 | 0.028 | 0.001 |
| Isoleucine, % | 0.075 | 0.141 | 0.003 |
| Leucine, % | 0.144 | 0.267 | 0.006 |
| Lysine, % | 0.038 | 0.070 | 0.001 |
| Methionine, % | 0.013 | 0.028 | 0.001 |
| Phenylalanine, % | 0.081 | 0.155 | 0.003 |
| Proline, % | 0.169 | 0.323 | 0.007 |
| Serine, % | 0.163 | 0.309 | 0.006 |
| Theonine, % | 0.069 | 0.127 | 0.003 |
| Tyrosine, % | 0.050 | 0.098 | 0.002 |
| Valine, % | 0.106 | 0.197 | 0.004 |
| Crude protein, % | 1.75 | 3.09 | 0.061 |
